# Adeno-associated virus type 2 infection activates caspase dependent and independent apoptosis in multiple breast cancer lines but not in normal mammary epithelial cells

**DOI:** 10.1186/1476-4598-10-97

**Published:** 2011-08-09

**Authors:** Samina Alam, Brian S Bowser, Michael J Conway, Mohd Israr, Apurva Tandon, Craig Meyers

**Affiliations:** 1 Department of Microbiology and Immunology, Pennsylvania State University College of Medicine, 500 University Drive, Hershey, PA 17033, USA; 2 Section of Infectious Diseases, Department of Internal Medicine, Yale University School of Medicine, New Haven, Connecticut, USA; 3 Department of Microbiology, Immunology and Pathology, Colorado State University, Fort Collins, CO 80523, USA

**Keywords:** Adeno-Associated Virus Type 2, AAV2, Breast cancer, Pro-apoptotic therapeutics, Apoptosis, Cell cycle, Rep proteins, c-Myc

## Abstract

**Background:**

In normal cells proliferation and apoptosis are tightly regulated, whereas in tumor cells the balance is shifted in favor of increased proliferation and reduced apoptosis. Anticancer agents mediate tumor cell death via targeting multiple pathways of programmed cell death. We have reported that the non-pathogenic, tumor suppressive Adeno-Associated Virus Type 2 (AAV2) induces apoptosis in Human Papillomavirus (HPV) positive cervical cancer cells, but not in normal keratinocytes. In the current study, we examined the potential of AAV2 to inhibit proliferation of MCF-7 and MDA-MB-468 (both weakly invasive), as well as MDA-MB-231 (highly invasive) human breast cancer derived cell lines. As controls, we used normal human mammary epithelial cells (nHMECs) isolated from tissue biopsies of patients undergoing breast reduction surgery.

**Results:**

AAV2 infected MCF-7 line underwent caspase-independent, and MDA-MB-468 and MDA-MB-231 cell lines underwent caspase-dependent apoptosis. Death of MDA-MB-468 cells was marked by caspase-9 activation, whereas death of MDA-MB-231 cells was marked by activation of both caspase-8 and caspase-9, and resembled a mixture of apoptotic and necrotic cell death. Cellular demise was correlated with the ability of AAV2 to productively infect and differentially express AAV2 non-structural proteins: Rep78, Rep68 and Rep40, dependent on the cell line. Cell death in the MCF-7 and MDA-MB-231 lines coincided with increased S phase entry, whereas the MDA-MB-468 cells increasingly entered into G2. AAV2 infection led to decreased cell viability which correlated with increased expression of proliferation markers c-Myc and Ki-67. In contrast, nHMECs that were infected with AAV2 failed to establish productive infection or undergo apoptosis.

**Conclusion:**

AAV2 regulated enrichment of cell cycle check-point functions in G1/S, S and G2 phases could create a favorable environment for Rep protein expression. Inherent Rep associated endonuclease activity and AAV2 genomic hair-pin ends have the potential to induce a cellular DNA damage response, which could act in tandem with c-Myc regulated/sensitized apoptosis induction. In contrast, failure of AAV2 to productively infect nHMECs could be clinically advantageous. Identifying the molecular mechanisms of AAV2 targeted cell cycle regulation of death inducing signals could be harnessed for developing novel therapeutics for weakly invasive as well as aggressive breast cancer types.

## Background

Breast cancer is the most prevalent cancer in the world and is the leading cause of cancer related death in women (411,000 annual deaths represent 14% of female cancer deaths) [1,2]. Breast cancer is also the most frequent cancer of women (23% of all cancers) [1]. Routine screening and early detection have reduced the incidence of breast cancer, but despite optimal treatment, about 30% of women with recurrent disease develop distant metastases [3]. Although multiple chemotherapeutic strategies are currently in use for the treatment of breast cancer [4], active treatment of patients is determined by multiple factors such as the hormone-dependency of the cancer [5], activation of specific oncogenes [6], invasiveness and metastases [7], subsequent drug resistance [8-10] and the risk of potential toxicities with repeated therapy [4,11]. Many patients are also subjected to combination drugs, as no single agent offers a clear survival advantage over another [4]. In addition, reliable biomarkers correlating response to chemotherapy and survival have not been clearly defined [12]. As such, there is a clinical need for breast cancer therapeutics which potently target malignant cells resultant with identifiable biomarkers, independent of the type of breast cancer profile presented by the patient.

We have recently reported that the non-pathogenic, tumor suppressive human Adeno-Associated Virus Type 2 (AAV2) induced apoptosis in both low and high-grade Human Papillomavirus (HPV) positive cervical cancer cell lines but not in normal keratinocytes [13]. AAV2 induced cell death correlated with the expression of AAV2 non-structural Rep proteins and culminated in DNA laddering and caspase-3 activation/cleavage [13], both established hallmarks of apoptosis [14]. Since AAV2 induced apoptosis also coincided with increased S phase entry in HPV/AAV2 co-infected cells, our studies further suggested that coordinate manipulation of both cell-cycle and apoptosis pathways by AAV2 has the potential to suppress growth and proliferation of cervical cancer cells [13]. Our work further provides a molecular platform supporting earlier studies which suggested that AAV2 seropositivity is negatively correlated with the development of cervical cancer [15].

AAV2 has been shown to suppress DNA replication and oncogenicity [16] of a number of viruses including adenovirus [17], herpesvirus [18], pox virus [19] and human papillomavirus (HPV) [20]. The AAV2 encoded non-structural Rep78 protein has been shown to inhibit *in vitro *cellular transformation mediated by papillomaviruses [21-24] and has been mapped to the ability of its Rep proteins to downregulate transcription from the papillomavirus early promoters [23,25,26]. Rep78 also antagonizes expression and activity of cellular tumor suppressors abrogated by adenovirus infections, such as p53 [27], pRb [28] as well as cell cycle modulators such as E2F [29], thereby curbing cell growth and proliferation. A recent report also demonstrated the ability of AAV2 to induce a moderate degree of caspase activation during adenovirus coinfection [30]. In animal models, AAV2 was shown to suppress the growth of adenovirus and herpesvirus induced tumors [31,32]. The tumor suppressive functions of AAV2 are not restricted to virus-virus interactions as AAV2 also suppressed cells in culture transformed by activated oncogenes [33], selectively killed carcinogen-treated cells [19,34] and targeted uncontrolled cellular proliferation rates by implementing cell cycle blocks, growth arrest and differentiation [35-40]. These studies and our published report [13] cumulatively led us to hypothesize that AAV2 also has the potential to mediate death of breast cancer cells.

In the current study, we investigated the ability of AAV2 to infect and induce apoptosis in a variety of human breast cancer derived cell lines, representing a range of breast cancer types graded upon hormone dependency and aggressiveness: the luminal MCF-7 (ERα-positive) are weakly invasive and do not normally metastasize to bone [41]; MDA-MB-468 (ERα-negative) are weakly invasive and non-metastatic and were derived from human breast adenocarcinoma [42]; and MDA-MB-231 (ERα-negative) are highly invasive, basal cell line [43] derived from metastatic (pleural effusion), infiltrating ductal breast carcinoma [44]. The MDA-MB-231 cell line is highly metastatic which forms tumors when injected into the mammary fat pads of immunocompromised mice [44]. As controls, we used primary normal human breast epithelial cells (nHMECs) isolated and cultured from breast tissue samples from patients undergoing breast reduction surgery. AAV2 infection induced caspase-independent apoptosis in the MCF-7 and caspase-dependent apoptosis in the MDA-MB-468 and MDA-MB-231 cell lines. In contrast, normal human mammary epithelial cells (nHMECs) were unaffected upon infection with AAV2. Cell death of breast cancer cells coincided with active AAV2 genome replication and differential expression of AAV2 non-structural Rep proteins: Rep78, Rep68 and/or Rep40 but not Rep52. AAV2 induced cell death of the MCF-7 and MDA-MB-231 lines was characterized by an increase in the number of cells with S phase DNA content whereas apoptotic MDA-MB-468 lines infected with AAV2 were increasingly arrested in the G2 phase of the cell cycle. Decreased cell viability in all three cell lines infected with AAV2 was characterized by upregulated expression of c-Myc and Ki-67, both proteins which are markers of proliferation and c-Myc is also a pro-apoptotic protein. Our results portray activation of distinct cell cycle and apoptosis pathways which culminate in cell death of all three breast cancer derived lines. Since the choice of specific therapeutics for the treatment of breast cancer is often determined by the aggressiveness of the cancer presented by individual patients, our studies suggest that AAV2 could have universal applicability for derivation of common yet novel therapeutics for a range of breast cancer types.

## Methods

### Cell lines

The MCF-7 [41], MDA-MB-468 [42] and MDA-MB-231 [43] cell lines have been previously described. Cells were maintained in monolayer culture with E medium containing 5% fetal bovine serum. Primary normal human breast epithelial cells (nHMECs) were cultured from tissues of patients undergoing breast reduction surgery, using trypsin digestion at 37°C as previously described [45]. The nHMECs were maintained in monolayer cultures without feeder cells, with 154 medium (Cascade Biologics, Portland, Oreg.), supplemented with antibiotics (Cascade Biologics) and human keratinocyte growth supplement (Cascade Biologics). Patient breast tissue samples were obtained using an Institutional Review Board approved exemption status (Penn State University College of Medicine, IRB No. 25284NHR).

### Chemcials

Staurosporine, Cycloheximide and Tumor Necrosis Factor α were purchased from Sigma.

### Viral stocks

AAV2 stocks were prepared in our laboratory using the whole-cell lysate method, by banding twice on CsCl gradients and determination of infectious titers as we have described previously [13]. The AAV2 virus stocks were tested for residual contamination with helper adenovirus using the following methods. A 0.5 μl aliquot from the CsCl gradient fractions was used to infect 293 cells in 24-well dishes. The AAV2 positive fractions exhibiting low cytopathic effects were pooled, and heated at 56°C for 30 min. The AAV2 stocks were then serially diluted and further used to infect 293 cells in 60-mm dishes and the presence of residual contaminating adenovirus was visualized using plaque assays.

### Cell synchronization and infection of cells

The MCF-7, MDA-MB-468, MDA-MB-231 and nHMEC cell lines were grown to approximately 80% confluence, trypsinized, and plated at a density of 1 × 10^6 ^cells in 100-mm plates in E medium. After plating, cells were incubated between 10 and 12 h, at which time about two-thirds or more of the cells are synchronized at the G1 phase (without the addition of exogenous chemicals) as previously described [13]. This time point was designated time zero. The medium was aspirated from the plates, and infections were carried out using AAV2 at a multiplicity of infection (MOI) of 0.02 (using AAV2 MOIs of 10, 20, 30 and 100 yielded similar results). The infections were performed by diluting the AAV2 stocks into 1 ml of E medium without serum and used for infection of cells. Mock infections were performed using 1 ml of E medium without serum. Plates were incubated at 37°C for 2 h with intermittent swirling. At the end of the incubation, residual medium was aspirated from the plates and replaced with 10 ml of fresh E medium supplemented with serum. Virus infected and mock infected cells were collected at t = 0 and day 1 through day 7 post-infection with AAV2. Using this method, the nHMECs could be passaged and viably cultured between 5 to 7 days due to their inherent primary cell growth characteristics. Both mock-infected and AAV2-infected cell samples were trypsinized, inactivated with the addition of serum, pelleted, and stored at -70°C until further manipulations. Additionally, on day 2 and day 5 (when cells were approximately 80% confluent), both mock and AAV2 infected cells were passaged at a ratio of 1:2 and plated in fresh E medium.

### Southern blot analysis of AAV2 DNA replication

A 5 μg aliquot of the isolated low molecular weight DNA was separated by electrophoresis in a 0.8% TAE agarose gel followed by transferring to GeneScreen Plus membranes (New England Nuclear Research Products, Boston, MA), as previously reported [46]. Samples were probed with ^32^P-labeled total AAV2 genomic DNA probe generated by random primer extension using methods previously described [46].

### Transfection assays

Cells were transfected using the calcium phosphate method as previously described [47]. Both *N*,*N*-bis(2-hydroxyethyl)-2-aminoethane-sulfonic acid (BES) and CaCl_2 _were purchased from Sigma-Aldrich. Briefly, a stock solution of 2.5 M CaCl_2 _was prepared and filter sterilized through a 0.22 μm filter, and stored at -20°C. A stock solution of 2× BES-buffered saline (2× BBS) was prepared containing 50 mM BES, 280 mM NaCl, and 1.5 mM Na_2_HPO_4_. The pH was adjusted to pH 6.95 with HCl, filter sterilized through a 0.22 μm filter and stored at -20°C. Cells were seeded 24 h prior to transfection using 10^6 ^cells per 100-mm dish in E-medium containing 5% FBS without the addition of antibiotics. The mixture for transfecting cells was prepared as follows: 30, 60 and 100 μg of the plasmid containing the full-length AAV2 genome was added to deionized water up to a volume of 450 μl, followed by addition of 50 μl of 2.5 mM CaCl_2_. Finally, 500 μl of 2× BBS was added to this mixture, mixed gently, followed by incubation at room temperature for 10-20 min. To determine the transfection efficiency, 30 μg of a green fluorescent protein (GFP) expression vector (Clontech) was transfected alone or co-transfected with the AAV2 genome into cells and used as a surrogate marker for delivery of the unlabeled AAV2 plasmid DNA where indicated. Transfection controls were essentially cells treated with the calcium phosphate precipitate without the addition of plasmid DNA. The 1 ml DNA suspension was mixed by gently inverting and added to the 100 mm culture dishes dropwise, followed by swirling the plates to evenly distribute the DNA precipitate. Following 24 hr of incubation, the medium was removed and cultures were rinsed twice using E-medium without serum and finally replaced with E-medium supplemented with 5% FBS without antibiotics and incubation was further continued for 24 hr. These samples were designated as the 48 hr samples post-transfection. Another set of cells was further incubated for 5 days and designated as the 7 day samples post-transfection. At the end of the designated incubation times cells were harvested by trypsinization, washed with phosphate-buffered saline (PBS) and fixed in 70% ethanol and prepared for FACS analysis using protocols described below.

### Preparation of total cellular protein extracts and Western blotting

Total protein extracts were prepared from the monolayer cultures and quantitated using the Lowry Method as previously described [13,48]. For detecting cell cycle proteins, a total of 60 μg whole cell extract was used in Western blots to determine expression of p21^WAF1^, p27^KIP1 ^and p16^INK4^. To detect pRb and p53 protein expression, 30 μg of whole cell extracts were used. Protein extracts were applied to sodium-dodecyl sulfate (SDS)-polyacrylamide (SDS-PAGE) gel (acrylamide/bisacrylamide ratio, 30:0.8). Gel compositions for resolving various proteins are as follows. A 12% gel was used to detect p21^WAF1^, 15% gel for p16^INK4^, 10% gel for p27^KIP1^, and 7.5% SDS-PAGE gel for pRb and p53. Polyclonal antibodies against p21^WAF1^, p27^KIP1^, p16^INK4 ^and pRb (Santa Cruz) were each used at a dilution of 1:2000 to detect the respective proteins, and have been described previously [13,48]. A monoclonal antibody against p53 (Oncogene) was used at a dilution of 1:1000 for detecting the protein and has been described previously [13,48].

To detect c-Myc and Ki-67 protein expression, 60 μg of whole cell extracts were used. Monoclonal antibodies against c-Myc (Santa Cruz) were used at a dilution of 1:500 and polyclonal antibodies against Ki 67 (Santa Cruz) were used at a dilution of 1:2000. For detecting involucrin, K-10 and K-14 expression, 5 μg of whole cell extract was used. The mouse monoclonal involucrin antibody (clone SY; Sigma) and K-10 antibody (BioGenex) were used at a dilution of 1:7000. The mouse monoclonal antibody (Sigma) was used at a dilution of 1:3000.

To detect apoptosis regulatory proteins, 60 μg of whole cell extracts were used. To detect the pro-caspase form of caspase-3, proteins were resolved in a 10% SDS-PAGE gel and detected with caspase-3 rabbit monoclonal antibody (Cell Signaling Technology) and used at a dilution of 1:1000. To detect the 19 kDa and 17-kDa cleaved caspase-3 forms, proteins were resolved in a 15% SDS-PAGE gel and detected with rabbit polyclonal antibody against cleaved caspase-3 (Cell Signaling Technology) and used at a dilution of 1:1000. To detect the pro-caspase form of caspase-6, proteins were resolved in a 10% SDS-PAGE gel and to detect the cleaved form of caspase-6, proteins were resolved in a 15% SDS-PAGE gel and detected with a rabbit polyclonal antibody (Cell Signaling Technology) used at a dilution of 1:1000. To detect both the pro- and cleaved forms of caspase-7, caspase-8 and caspase-9, proteins were resolved in a 10% SDS-PAGE gel. Caspase-7 was detected with a mouse monoclonal antibody (Cell Signaling) and used at a dilution of 1:1000. Caspase-8 was detected with a mouse monoclonal antibody (Alexis Biochemicals) and used at a dilution of 1:2000. Caspase-9 was detected with a rabbit polyclonal antibody (Cell Signaling) and used at a dilution of 1:1000. To detect the pro- and cleaved forms of PARP, proteins were resolved in a 7.5% SDS-PAGE gel and detected with a rabbit monoclonal antibody (Cell Signaling) and used at a dilution of 1:1000. Proteins were detected using enhanced chemiluminescence (ECL) method (Perkin Elmer) as per manufacturer's instructions.

### Cell viability assay and flow cytometric analysis of DNA content

MCF-7, MDA-MB-231, MDA-MB-468 cultures were synchronized and infected with AAV2 as described above. At each time point, cell samples were collected at t = 0 and days 1 through 7. On day 2 and day 5, both mock and AAV2 infected cells were passaged 1:2 as described above. Both mock and AAV2 infected cell samples were trypsinized, inactivated with the addition of serum, and counted using trypan blue exclusion using standard protocols.

For performing flow cytometry, mock and AAV2 infected cells were prepared for analysis as previously described [48]. Briefly, MCF-7, MDA-MB-231 and MDA-MB-468 cells were synchronized and infected with AAV2 as described above. Mock and AAV2 infected cells were sampled at t = 0 and from day 1 through day 7, with passaging on day 2 and day 5 as described. On each day, cells were harvested by trypsinization, washed with PBS, fixed in 70% ethanol, and stored at -20°C. The fixed cells were washed in PBS and then resuspended in PBS containing 0.1% Triton X-100 (Sigma), 200 μg/ml DNase-free RNase A (Boehringer Mannheim), and 100 μg/ml of propidium iodide (Sigma) for 30 min at 37°C. Flow cytometric analysis of 10^6 ^cells was carried out in a fluorescence-activated cell sorter (FACS), and the percentages of cells in the G1, S and G2/M phases of the cell cycle were determined using the Cell Quest program of Becton Dickinson. Data were analyzed with the Mod Fit LT program.

## Results

### AAV2 infection induces apoptosis in the breast cancer derived cell lines but not in normal breast epithelial cells

Multiple studies, including our published report [13], have documented the anti-proliferative nature of AAV2. We further wanted to examine the possibility that AAV2 infection could also suppress growth and proliferation of breast cancer derived cells. In the current study, we used three different human breast cancer derived cell lines representing a range of breast cancer types: MCF-7 (ER^+^), MDA-MB-468 cells (ER^-^) and MDA-MB-231 (ER^-^) cells. As controls, we used primary normal human breast epithelial cells (nHMECs) isolated and cultured from breast tissue samples from patients undergoing breast reduction surgery.

**Figure 1:**
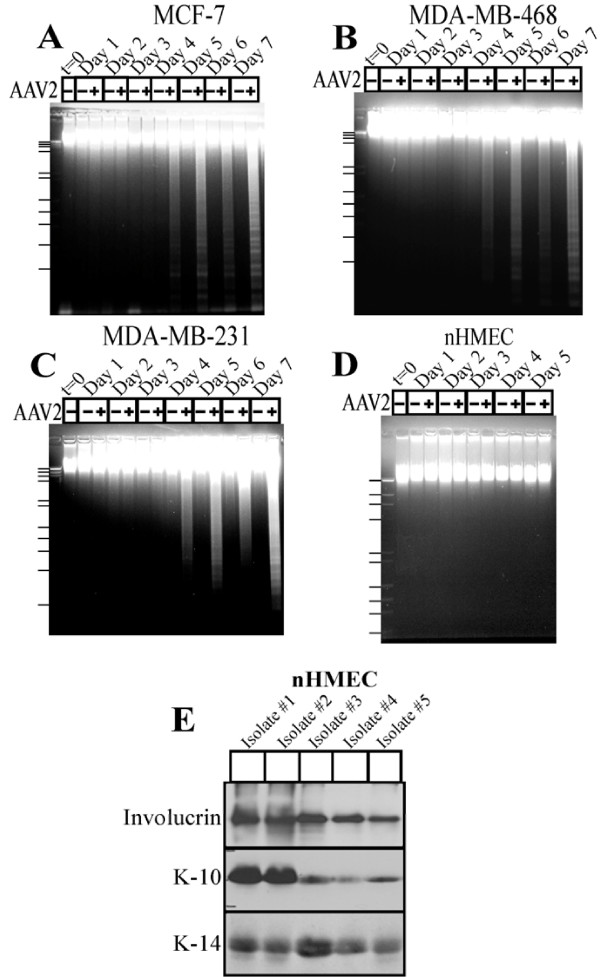
**AAV2 induced DNA laddering/degradation in multiple breast cancer cell lines**. (A) MCF-7, (B) MDA-MB-468, (C) MDA-MB-231 and (D) nHMECs monolayer cultures were synchronized in G1, followed by infection with AAV2 at an MOI of 0.02. Cell pellets were collected each day over a seven day period for the breast cancer cell lines, and over a five day period for the nHMECs. The breast cancer cell lines were passaged 1:2 on day 2 and day 5, and on day 2 for nHMECs. DNA laddering assays were performed by isolating low-molecular weight DNA using protocols described herein. Twenty micrograms of DNA were resolved in a 1% agarose Tris-Borate-EDTA gel and stained with ethidium bromide. Results shown are representative of three individual experiments. t, time; +, AAV2 infected; -, control. DNA ladders are indicate the following base-pair fragments starting at the top: 23941, 9416, 6557,4361, 2322, 2027, 1353, 1073, 872, 603, 310. (E) nHMECs cultured from multiple patient breast tissue biopsies were used to prepare whole cell extracts. Five micrograms of the extract was used for western blot analysis. Cytokeratin markers Involucrin, K10 and K14 were detected using antibodies as described in Materials and Methods.

The MCF-7, MDA-MB-468, MDA-MB-231 and nHMEC cells were synchronized as described in Materials and Methods, when approximately two-thirds or more of the cells were in the G1 phase of the cell cycle [13,48]. Under our culture conditions, this is the maximum level of synchronization that we normally achieve with or without the addition of exogenous chemicals. Synchronized cultures were infected with AAV2 using an MOI of 0.02 (using AAV2 MOIs of 10, 20, 30 and 100 yielded similar results). Both AAV2-infected and mock-infected cells were grown to 80% confluence (day 2), at which time the cells were passaged at a ratio of 1:2, followed by a period of further growth and passaging again at a 1:2 ratio on day 5. On day 5, each of the AAV2 infected breast cancer cell lines showed growth retardation, and eventually culminated in complete apoptotic cell death as evidenced by visualizing the DNA laddering in the MCF-7 and MDA-MB-468 cells (Figure 1A and 1B), and a mixture of DNA laddering and non-specific DNA degradation in the MDA-MB-231 cells (Figure 1C). In contrast, AAV2 infection of nHMECs did not induce DNA laddering or degradation (Figure 1D), but both control and AAV2 infected nHMECs eventually underwent senescence. Due to their inherently primary growth characteristics in culture, nHMECs isolated from patient biopsies have a limited life-span in culture. The DNA laddering experiments were performed with a batch of nHMECs which was limited to 5 days in culture. The inability of AAV2 to induce DNA laddering or degradation in the normal epithelial cells tested was reproducible in multiple batches of nHMECs isolated from individual patients. Using western blot analysis we also confirmed the epithelial origin of the different isolates of nHMECs by detecting the expression of cytokeratins which are known to be expressed in the normal breast epithelial cells, such as K-10 [49], K-14 [50] and involucrin [51] (Figure 1E). Qualitative differences between the different batches of nHMECs isolated did not affect their ability to be infected with AAV2 as was further verified by performing other assays as described elsewhere in this manuscript. We have similarly reported that AAV2 also specifically targets HPV positive cervical cancer cells representing both low and high cancer grades but not primary human keratinocytes [13].

**Figure 2:**
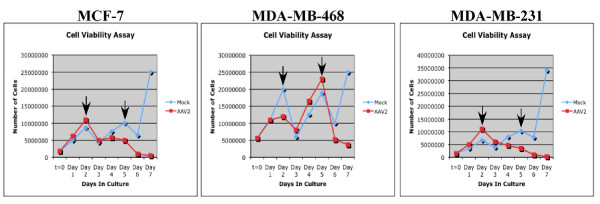
**Cell viability as a measure of AAV2 induced cell death in the breast cancer cell lines**. (A) MCF-7, (B) MDA-MB-468 and (C) MDA-MB-231 cells were infected with AAV2. Trypan blue exclusion was used to determine the growth inhibitory effect of AAV2 on the monolayer cultures of the different breast cancer cell line cultures. Results shown are representative of three individual experiments. t = time. Arrows indicate days when the cells were passaged at a ratio of 1:2.

Apoptosis induction in the three breast cancer lines tested was also portrayed as a steady decrease in cell viability over the 7 day period post-infection with AAV2 (Figure 2). On day 8, we observed a 100% death of each of the AAV2 infected cell lines (data not shown). These results suggest the specificity of AAV2 targeted cell killing of the breast cancer cells regardless of breast cancer grade. Since complete cell death was accomplished using an initial MOI of 0.02, this meant that on average 1 out of 50 cells were infected. Therefore, the mechanism of cell killing was potentially mediated by a "by-stander" type of effect, in which one cell undergoing cell death releases cytotoxic metabolites, cellular proteins, or in this study, perhaps AAV2 encoded proteins, which are able to spread to surrounding non-infected cells through cellular gap junctions, and mediate cell killing, as has been previously described [52,53].

### AAV2 infection of breast cancer cells results in Rep protein expression and genome replication which culminates in apoptosis induction

**Figure 3:**
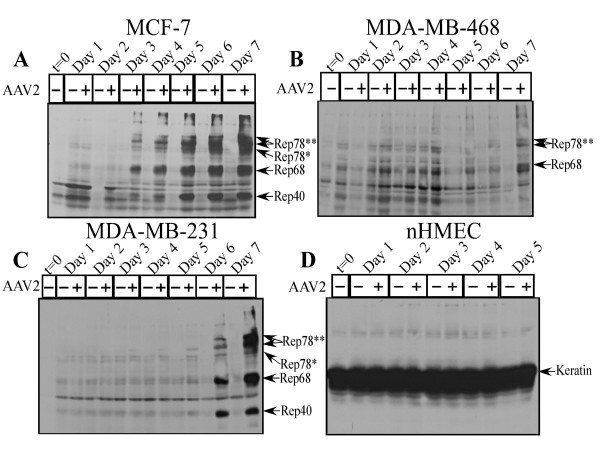
**AAV2 induction of apoptosis/cell death in the different breast cancer lines correlates with Rep protein expression**. For detecting Rep proteins in Western blots, total protein extracts were prepared as we have previously described [13,48]. Sixty micrograms of total protein extracts from AAV2 infected and control (A) MCF-7, (B) MDA-MB-468, (C) MDA-MB-231 and (D) nHMECs monolayer cultures were resolved in a 7.5% SDS-polyacrylamide gel electrophoresis gel and detected with an AAV2 Rep-specific antibody (Progen). Results shown are representative of three individual experiments. t, time; +, AAV2 infected; -, control. Double asterisks (**) indicate dimer form of protein, single asterisk (*) indicates the monomer form.

**Figure 4:**
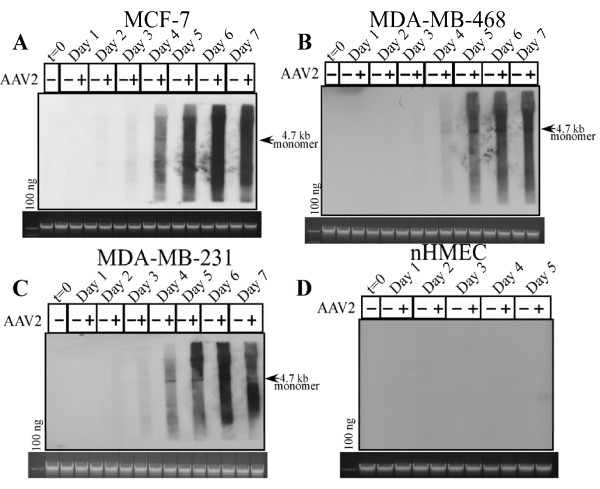
**Southern blot analysis of AAV2 genome replication in breast cancer cell lines**. Southern blot analysis to detect 4.7 kb AAV2 replicative form monomer representing active genome replication in AAV2 infected (A) MCF-7, (B) MDA-MB-468, (C) MDA-MB-231 breast cancer cells and (D) nHMECs. A total of 5 μg of total DNA was then detected with AAV2 genomic DNA as probe as previously described [46]. One hundred nanograms of total DNA isolated from cells was used as loading control (bottom). Results shown are representative of three individual experiments. t, time; +, AAV2 infected; -, control.

AAV2 encodes four non-structural proteins: Rep78 and Rep68, which regulate multiple viral functions including DNA replication and transcription; and Rep52 and Rep40, which are involved in packaging of viral genomes into capsids [16]. The Rep78, Rep68 and Rep40 proteins were differentially expressed in the multiple breast cancer lines infected with AAV2 (Figure 3A, B and 3C) but Rep52 expression could not be determined in any of the virus infected cell lines. In contrast, AAV2 infected nHMECs did not express any Rep proteins (Figure 3D). The kinetics of Rep protein expression and their relative abundance varied between the different breast cancer lines. While Rep78 and Rep68 expression was clearly detected in AAV2 infected MCF-7 cells beginning on day 3 and continuing up to day 7 (Figure 3A), in the AAV2 infected MDA-MB-231 cells only Rep78 expression was barely detectable on day 3, with progressive increase in both Rep78, Rep68 and Rep40 expression at later time points during the same time-course (Figure 3C). Rep protein expression on day 3 preceded apoptotic DNA laddering in the MCF-7 cells, and DNA laddering/degradation in MDA-MB-231 cells which was observed on day 4, but increased in subsequent days (compare Figure 3 to Figure 1). Our results also agree with previously published reports which showed that the expression of Rep78 alone was shown to be sufficient for induction of apoptosis in HL60 cells in vitro [54]. In contrast to the other two breast cancer cell lines, in the AAV2 infected MDA-MB-468 cells, only Rep78 expression could be determined with clarity on day 6 and day 7 (Figure 3B), with potentially Rep68 also being expressed (Figure 3B). The antibody used for detecting the Rep proteins bound to a cross-reacting band in the control lanes of MDA-MB-468 cells which resolved near to Rep68 in the AAV2 infected samples (Figure 3B), but which was less intense than the Rep68 band. In MDA-MB-468 cells, apoptotic DNA laddering was observed on day 4 (Figure 1B), but Rep78 protein was not clearly detected until days 6 and 7 (Figure 3B). Since AAV2 genome replication was detected as early as day 4 in the virus infected cells (Figure 4B), it is possible that under these conditions, in the MDA-MB-468 cells, low levels of Rep protein were expressed earlier than day 6 but expression was at the limits of detection using the techniques used.

We consistently detected the dimer form of Rep78 in all three cell lines (Figure 3), which is not uncommon since others have also reported finding the high molecular weight Rep78 complexes, which are essentially Rep-Rep protein aggregates containing two to six Rep proteins which persist in sodium dodecyl sulfate (SDS) [55,56]. We have also detected these high molecular weight Rep78 complexes in HPV/AAV2 co-infected cervical cancer cells undergoing apoptosis [13].

To rule out the possibility that the failure of AAV2 infected nHMECs to express Rep proteins was due to the inability of the virus to infect the normal breast epithelial cells, we performed Southern blot analysis to detect the status of AAV2 DNA replication. The 4.7 kb replicative form DNA monomer was detectable in the three AAV2 infected breast cancer cell lines tested (Figure 4A-C), but not in the nHMECs (Figure 4D). The inability of AAV2 to replicate in nHMECs was also confirmed by the absence of Rep protein expression in these cells (Figure 3D). Thus, the failure of AAV2 to productively infect nHMECs could lead to failure of Rep proteins being expressed and subsequently undergo apoptosis.

**Figure 5:**
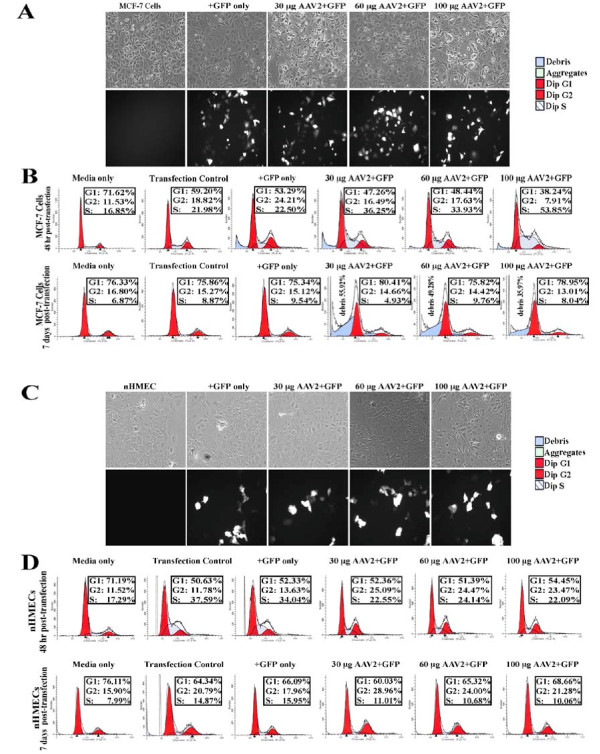
**Calcium-phosphate transfection of the cloned AAV2 genome into MCF-7 cells and nHMECs**. (A) MCF-7 cells. Untransfected cells, GFP-only controls (+GFP), and GFP vector and AAV2 genome co-transfected cells (+GFP/+AAV2). All images were captured with a 20× objective. (B) MCF-7 cells. Fluorescent Activated Cell Sorting (FACS) Analysis of transfected cells analysed at 48 h and 7 days post-transfection. Percentages of different cell cycle phases are represented in the G1, S and G2 fractions. Percentage denoting "Debris" is indicative of DNA damage induced cell death/loss of membrane integrity. (C) nHMECs. Untransfected cells, GFP-only controls (+GFP), and GFP vector and AAV2 genome co-transfected cells (+GFP/+AAV2). All images were captured with a 20× objective. (D) nHMECs. Fluorescent Activated Cell Sorting (FACS) Analysis of transfected cells analysed at 48 h and 7 days post-transfection. Percentages of different cell cycle phases are represented in the G1, S and G2 fractions.

To address the possibility that AAV2 failed to productively infect nHMECs and induce apoptosis, we analyzed the effect of physically delivering the full-length AAV2 genome into nHMECs using standard calcium phosphate DNA co-precipitation and transfection protocols. We have previously utilized this method to show that transfection of the AAV2 genome into HPV positive cells resulted in cell death [13], whereas normal human keratinocytes similarly transfected were unaffected [13]. In the current study, we transfected 30, 60 and 100 μg of the full-length cloned AAV2 genome into the nHMECs and MCF-7 cells. The same AAV2 clone was also used for production of the AAV2 virus stocks. To determine the transfection efficiency, we also co-transfected 30 μg of a green fluorescent protein (GFP) expression vector (Clontech) as a surrogate marker for the delivery of the unlabeled AAV2 genome into the two cell types. In our hands, the efficiency of the transfection protocol performed with 30 μg of the GFP expression vector alone routinely resulted in an average of 20% of each cell type being GFP positive (Figure 5A and 5C), which is within the efficiency range reported for keratinocytes in published studies [57,58]. At 48 h post-transfection, both the MCF-7 cells and nHMECs co-transfected with the AAV2 genome/GFP expression vector expressed numbers of GFP-positive cells that were approximately equal to the number of cells in each respective type expressing GFP alone (Figure 5A and 5C). At this time point, growth/adherence of the AAV2-transfected MCF-7 cells and nHMECs was similar to the GFP only control transfected cells (Figure 5A). These results are in contrast to our earlier studies which clearly showed cell death of the AAV2-transfected HPV cells at 48 h post-transfection but not the primary human keratinocytes [13]. An alternative possibility was that AAV2 transfection of breast epithelial cells result in delayed cellular effects which required incubations beyond the 48 h time-point post-transfection. To further determine whether AAV2 transfection into the MCF-7 cells and nHMECs resulted in identifiable early and late changes, we performed cell cycle analysis. We have previously reported the ability of AAV2 infection to modulate the cell cycle machinery in cervical cancer cells at both early and late times post-infection [13,48]. We determined the percentage distribution of the AAV2 genome transfected MCF-7 and nHMECs in G1, S, G2 and M phases of the cell cycle at 48 h and 7 days post-transfection. As mentioned above, nHMECs could be viably cultured up to 7 days. At 48 hr post-transfection, AAV2 transfected MCF-7 cells showed a sustained increase in the percentage of cells with S phase DNA content compared to control transfected- as well as GFP only-transfected cells (Figure 5B). This observation was significant, since we have previously shown that AAV2 infected HPV positive cells increasingly entered into S phase which was followed by Rep protein expression and apoptosis induction [13] and was also shown to be true for AAV2 infected MCF-7 cells (discussed below). In contrast, at 7 days post-transfection, the percentage of MCF-7 cells in S phase was similar to controls (Figure 5B), but an increased percentage of dead cells was indicated by the appearance "debris", essentially composed of damaged cell membranes which weakly stain with propidium iodide as a result of DNA loss. Therefore, detection of damaged cellular membranes was a measurable consequence of AAV2 induced cell death. In contrast, at 48 h post-transfection, AAV2 transfected nHMECs displayed S phase fraction of cells which was similar to control transfected cells (Figure 5D), and these levels did not change after 7 days post-transfection (Figure 5D). Additionally, AAV2 induced cellular membrane damage ("debris") was not observed at either 48 h or 7 day time-points post-transfection (compare Figure 5B with Figure 5D). Our results cumulatively suggest that although AAV2 is capable of infecting nHMECs it is unable to establish a persistent infection. These results essentially duplicated our earlier observations that AAV2 was unable to establish a persistent infection in primary keratinocytes [13]. The observed differences are potentially due to sensitivities of the cancer lines to AAV2 mediated gene transcription compared with primary cells. To rule out the possibility that qualitative differences between the different batches of nHMECs isolated affected their ability to be transfected with AAV2, we performed the virus infection and genome transfection experiments with multiple batches of cells and obtained similar results.

**Figure 6:**
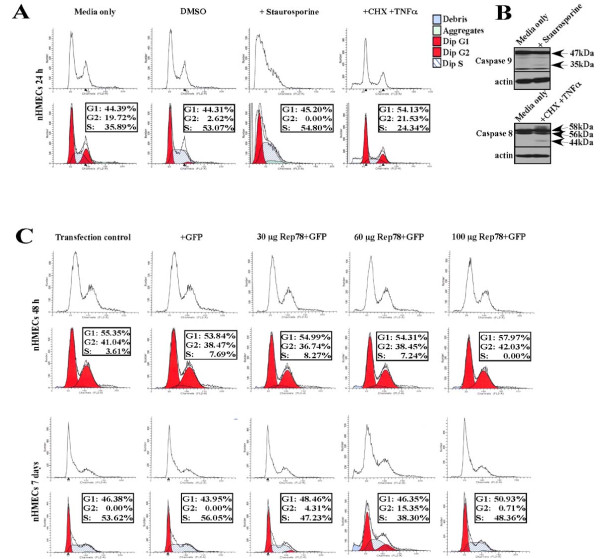
**Determining the ability of nHMECs to undergo chemically induced and Rep78 induced apoptosis**. (A) nHMECs were treated with either staurosporine (1 μM) to activate the instrinsic pathway, and a combination of cycloheximide (CHX) (10 μg/ml) and Tumor Necrosis Factor-Alpha (TNFα) (20 ng/ml) to activate the extrinsic pathway of apoptosis. Cells were treated for 24 h, harvested prepared for FACS analysis, and 10^6 ^cells were analyzed by flow cytometry. Top panel denotes histograms of the DNA distribution representing percentages of cells in the G1, S and G2 phases of the cell cycle denoted in the bottom panel, as determined using the Cell Quest program of Becton Dickinson. Data were analyzed with the Mod Fit LT program. (B) Staurosporine treatment induced cleavage of caspase-9 and CHX/TNFα treatment induced cleavage of caspase-8 as determined from western blot analysis. (C) Calcium-phosphate transfection of the cloned Rep78 expression construct under CMV promoter control into nHMECs. Untransfected control, GFP-only controls (+GFP), and GFP vector and CMV-Rep78 construct co-transfected cells (+GFP/Rep78). Top panel denotes histograms of the DNA distribution representing percentages of cells in the G1, S and G2/M phases of the cell cycle denoted in the bottom panel, as determined using the Cell Quest program of Becton Dickinson. Data were analyzed with the Mod Fit LT program. Analysis of transfected cells analyzed at 48 h and 7 days post-transfection.

The inability of nHMECs to undergo apoptosis was of interest from a mechanistic point of view, since a potential concern was the possibility that the nHMECs had acquired modifications which rendered them resistant to apoptosis. To show that the cells were still sensitive to apoptotic signals, we treated the nHMECs with staurosporine (1 μM), which has been shown to activate the instrinsic pathway of apoptosis, and a combination of cycloheximide (CHX) (10 μg/ml) and Tumor Necrosis Factor-Alpha (TNFα) (20 ng/ml), which has been shown to activate the extrinsic pathway of apoptosis. Treatment with both staurosporine and CHX/TNFα resulted in increased accumulation of the sub-G1 cell population as shown in the FACS analysis, and which is an indicator of apoptosis (Figure 6A). Further, staurosporine mediated cleavage of caspase-9, a marker for the intrinsic, and CHX/TNFα mediated cleavage of caspase-8, a marker for the extrinsic pathway of apoptosis (Figure 6B). These results show that the inability of nHMECs to undergo AAV2 mediated cell death was not due to the acquisition of death resistance pathways.

We also analyzed the ability of transfected Rep78 protein expression construct to induce cell death of nHMECs. For these experiments, 30, 60 and 100 μg of the Rep78 expression vector under control of the CMV promoter (a kind gift of A. Marcello) [59] was transfected into nHMECs. We performed FACS analysis of the transfected cells both at 48 h and 7 days post-transfection. Neither early or late time points post-transfection showed the ability of Rep78 to induce cell death to any measurable degree (Figure 6C) (and compare Figure 5 with Figure 6). The inability of Rep78 to induce cell death of nHMECs could suggest the possibility that Rep78 alone is insufficient for death induction and perhaps the other Rep proteins need to be co-expressed for apoptosis to occur.

### AAV2 Rep protein expression results in activation of both caspase-dependent and -independent pathways of apoptosis induction in the multiple breast cancer lines

Apoptotic cell death occurs due to activation of two signaling pathways: first, the extrinsic, or receptor-mediated pathway, and secondly, the intrinsic or mitochondrial pathway [14]. Two groups of caspases characterize these pathways: the initiator (caspase-8, -9, and -10) and executioner (caspase-3, -6, -7) caspases. Initiator caspases cleave and activate executioner caspases, which in turn process cellular substrates which orchestrate the biochemical execution of cell death [60]. We have previously shown that AAV2 induction of apoptosis in HPV positive cervical cancer cells was correlated with caspase-3 activation/cleavage [13]. In the current study, it was of interest to determine whether AAV2 induction of apoptotic cell death in the three different breast cancer cell lines was due to activation of common pathways.

**Figure 7:**
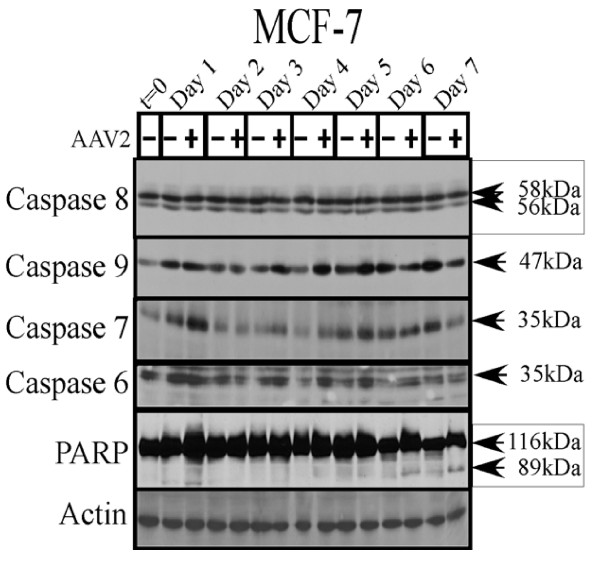
**AAV2 induction of apoptosis in MCF-7 cells results in PARP cleavage independent of caspase activation**. MCF-7 monolayer cell cultures were synchronized in G1, followed by infection with AAV2. Cell pellets were collected each day over a 7 day period. Cells were passaged 1:2 on day 2 and day 5. Detection of caspases and their cleavage/activation was performed by Western blotting. Total protein extracts were prepared as described previously [48]. Sixty micrograms of total protein extracts from AAV2 infected MCF-7 and control cells were resolved in SDS-polyacrylamide gel electrophoresis (PAGE) gels. To detect the 35 kDa pro-caspase form of caspase-6, proteins were resolved in a 10% SDS-PAGE gel and detected with a rabbit polyclonal antibody (Cell Signaling Technology). To detect both the pro- and cleaved forms of caspase-7, caspase-8 and caspase-9, proteins were resolved in a 10% SDS-PAGE gel. The 35 kDa pro-caspase form of caspase-7 was detected with a mouse monoclonal antibody (Cell Signaling). The 58 and 56 kDa forms of caspase-8 were detected with a mouse monoclonal antibody (Alexis Biochemicals). The 47 kDa pro-caspase form of caspase-9 was detected with a rabbit polyclonal antibody (Cell Signaling). To detect the pro- (116 kDa) and cleaved- (89 kDa) forms of PARP, proteins were resolved in a 7.5% SDS-PAGE gel and detected with a rabbit monoclonal antibody (Cell Signaling).

The endogenous caspase-3 protein is abundantly expressed in the MDA-MB-468 [61] and MBA-MB-231 [62] cell lines, but the MCF-7 cells do not express caspase-3 due to a 47-bp deletion within the corresponding gene [63]. To investigate which of the executioner caspases were cleaved upon AAV2 induced apoptosis in the breast cancer lines, we examined expression of caspases -3, -6 and -7 in total protein extracts. Since the MCF-7 cells do not express caspase-3, we examined the expression levels of other executioner caspases, caspase-6 and caspase-7, in mock and AAV2 infected cells (Figure 7). Neither executioner caspases -6 or -7 showed any significant or consistent changes in the levels of the full-length proteins (Figure 7), and their cleaved forms could not be detected (data not shown). Failure to observe cleaved forms of either executioner caspases -6 and -7 was unexpected since the AAV2 infected MCF-7 cells additionally displayed the 89 kDa cleaved fragment of PARP (Figure 7), a DNA repair enzyme which is generally inactivated by cleavage from one of the executioner caspases [64]. At the same time, apoptotic DNA laddering was observed in AAV2 infected cells (Figure 1A). It is possible that in AAV2 infected MCF-7 cells, other non-caspase dependent pathways of PARP inactivation potentially play a role in cell death, such as calpain-dependent PARP cleavage [65]. Furthermore, in our hands, apoptosis induction in AAV2 infected MCF-7 cells could not be correlated with cleavage of either initiator caspases -8 or -9 (Figure 7). Cumulatively, our results in the current study indicate that AAV2 targets caspase-independent pathway(s) of apoptosis in the virus infected MCF-7 cells.

**Figure 8:**
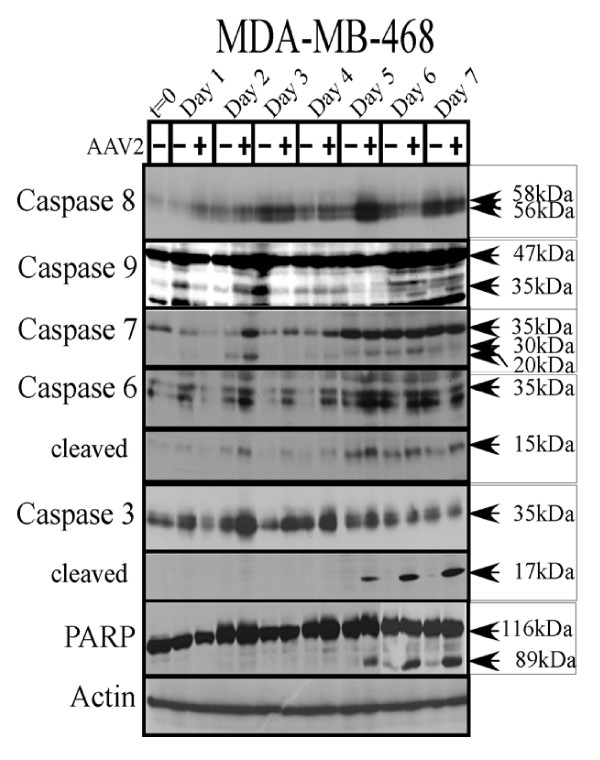
**AAV2 induction of apoptosis in MDA-MB-468 cells results in PARP cleavage following activation of caspases of the intrinsic pathway**. MDA-MB-468 monolayer cell cultures were synchronized in G1, followed by infection with AAV2. Cell pellets were collected each day over a 7 day period. Cells were passaged 1:2 on day 2 and day 5. Detection of caspases and their cleavage/activation was performed by Western blotting. Total protein extracts were prepared as described previously [48]. Sixty micrograms of total protein extracts from AAV2 infected and control MDA-MB-468 cells were resolved in SDS-polyacrylamide gel electrophoresis (PAGE) gels. To detect the 35 kDa pro-caspase form of caspase-3, proteins were resolved in a 10% SDS-PAGE gel and detected with caspase-3 rabbit monoclonal antibody (Cell Signaling Technology). To detect the17-kDa cleaved caspase-3 forms, proteins were resolved in a 15% SDS-PAGE gel and detected with rabbit polyclonal antibody against cleaved caspase-3 (Cell Signaling Technology). To detect the 35 kDa pro-caspase form of caspase-6, proteins were resolved in a 10% SDS-PAGE gel and to detect the 15 kDa cleaved form of caspase-6, proteins were resolved in a 15% SDS-PAGE gel and detected with a rabbit polyclonal antibody (Cell Signaling Technology). To detect both the pro- and cleaved forms of caspase-7, caspase-8 and caspase-9, proteins were resolved in a 10% SDS-PAGE gel. The 35 kDa pro-caspase form and the 30 kDa and 20 kDa cleaved forms of caspase-7 was detected with a mouse monoclonal antibody (Cell Signaling). Caspase-8 was detected with a mouse monoclonal antibody (Alexis Biochemicals). The 47 kDa pro-caspase and 35 kDa cleaved of caspase-9 was detected with a rabbit polyclonal antibody (Cell Signaling). To detect the pro- (116 kDa) and cleaved- (89 kDa) forms of PARP, proteins were resolved in a 7.5% SDS-PAGE gel and detected with a rabbit monoclonal antibody (Cell Signaling).

In contrast to the MCF-7 cells, both the initiator and executioner caspases were differentially cleaved in the AAV2 infected MDA-MB-468 cells. In these cells, AAV2 regulated PARP cleavage clearly correlated with cleavage of caspase-3 (Figure 8) and apoptotic DNA laddering (Figure 1B). AAV2 infected cells displayed the 17 kDa cleaved form of caspase-3 which was clearly visible starting on day 5 post-infection (Figure 8). In contrast to caspase-3, the 15 kDa cleaved form of caspase-6 was observed in both control and AAV2 infected MDA-MB-468 cells, although the levels of cleaved caspase-6 were higher in the virus infected cells compared with controls (Figure 8). Likewise, both the cleaved and uncleaved forms of caspase-7 were observed in these cells regardless of AAV2 infection, but their respective amounts could not be clearly correlated with AAV2 induced cell death (Figure 8). Therefore, PARP cleavage could be mediated by either caspase-3 or caspase-6 in the AAV2 infected MDA-MB-468 cells. In these cells, AAV2 induced apoptosis was further correlated with caspase-9 cleavage to its 35 kDa form but caspase-8 was unchanged (Figure 8). AAV2 mediated activation of caspase-9 suggested virus targeted disruption of mitochondrial functions and cytochrome *c *release. Our results clearly indicate AAV2 mediated targeting of the intrinsic pathways of apoptosis in the MDA-MB-468 cells.

**Figure 9:**
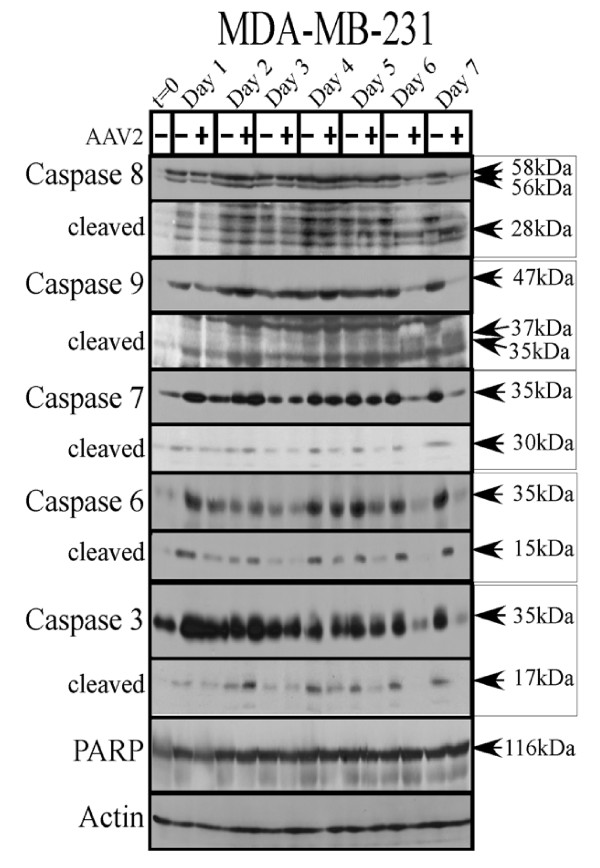
**AAV2 induction of apoptosis/cell death in MDA-MB-231 cells results in activation of caspases of both the intrinsic and extrinsic pathways but not PARP cleavage**. Left panel: MDA-MB-231 monolayer cell cultures were synchronized in G1, followed by infection with AAV2. Cell pellets were collected each day over a 7 day period. Cells were passaged 1:2 on day 2 and day 5. Detection of caspases and their cleavage/activation was performed by Western blotting. Total protein extracts were prepared as described previously [48]. Sixty micrograms of total protein extracts from AAV2 infected and control MDA-MB-468 cells were resolved in SDS-polyacrylamide gel electrophoresis (PAGE) gels. To detect the 35 kDa pro-caspase form of caspase-3, proteins were resolved in a 10% SDS-PAGE gel and detected with caspase-3 rabbit monoclonal antibody (Cell Signaling Technology). To detect the17-kDa cleaved caspase-3 forms, proteins were resolved in a 15% SDS-PAGE gel and detected with rabbit polyclonal antibody against cleaved caspase-3 (Cell Signaling Technology). To detect the 35 kDa pro-caspase form of caspase-6, proteins were resolved in a 10% SDS-PAGE gel and to detect the 15 kDa cleaved form of caspase-6, proteins were resolved in a 15% SDS-PAGE gel and detected with a rabbit polyclonal antibody (Cell Signaling Technology). To detect both the pro- and cleaved forms of caspase-7, caspase-8 and caspase-9, proteins were resolved in a 10% SDS-PAGE gel. The 35 kDa pro-caspase form and the 30 kDa/20 kDa cleaved forms of caspase-7 was detected with a mouse monoclonal antibody (Cell Signaling). The pro-caspase and cleaved 28 kDa form of caspase-8 was detected with a mouse monoclonal antibody (Alexis Biochemicals). The 47 kDa pro-caspase and 37 kDa/35 kDa cleaved forms of caspase-9 were detected with a rabbit polyclonal antibody (Cell Signaling). To detect the pro- (116 kDa) form of PARP, proteins were resolved in a 7.5% SDS-PAGE gel and detected with a rabbit monoclonal antibody (Cell Signaling).

In comparison with both the MCF-7 and MDA-MB-468 cell lines, the steady-state protein levels of both uncleaved and cleaved forms of caspases-3, -6 and -7 declined in the AAV2 infected MDA-MB-231 cells compared with mock infected cells (Figure 9). The MDA-MB-231 cells incidentally maintained high levels of the cleaved forms of these caspases in control cells (Figure 9). In the AAV2 infected cells, simultaneous decline of both uncleaved and cleaved forms of the three executioner caspases was potentially due to degradation by non-caspase proteases as has been previously suggested [66]. Specific cell death types have been attributed to non-caspase proteases in multiple studies [67]. Moreover, PARP cleavage was not detected in AAV2 infected MDA-MB-231 cells (Figure 9), further suggesting the failure of AAV2 to activate executioner caspases. The appearance of cleaved PARP is a measure of caspase activity [68]. A switch from apoptosis to activation of necrosis mediated cell death pathways could explain the failure to cleave PARP [69] as well as the appearance of non-specific DNA degradation mixed with the apoptotic DNA ladders (Figure 1C). In contrast to the executioner caspases, death of AAV2 infected MDA-MB-231 cells on day 6 and day 7 was correlated with cleavage of the initiator caspase-8 to its 28 kDa species and caspase-9 to 35 and 37 kDa proteolytic species (Figure 9). The AAV2 regulated cleavage of caspase-9 implicated disruption of mitochondrial functions and release of cytochrome *c*. On the other hand, the simultaneous cleavage of caspase-8 in the AAV2 infected cells indicated the activation of the extrinsic pathways of apoptosis. Cumulatively, our results suggests AAV2 mediated co-activation of the extrinsic and the intrinsic pathways of apoptotic cell death in the MDA-MB-231 cells. In addition, simultaneous triggering of non-caspase protease regulated cell death pathways cannot be ruled out. Therefore, activation of multiple cell death pathways could culminate in death of the MDA-MB-231 cells.

**Figure 10:**
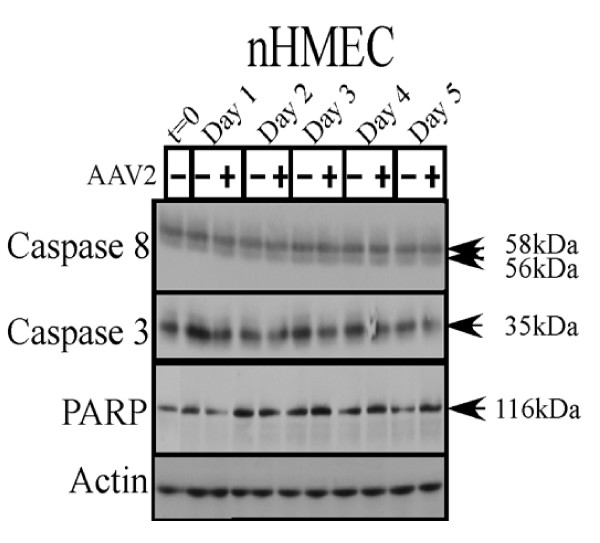
**AAV2 infection of nHMECs does not result in activation of caspases of either the intrinsic and extrinsic pathways of apoptosis**. nHMEC monolayer cultures were synchronized in G1 as described, followed by infection with AAV2. Cell pellets were collected each day over a 5 day period. Cells were passaged 1:2 on day 2. Detection of caspases and their cleavage/activation was performed by Western blotting. Sixty micrograms of total protein extracts from AAV2 infected and control nHMEC cells were resolved in SDS-polyacrylamide gel electrophoresis (PAGE) gels. To detect caspase-8 proteins were resolved in a 10% SDS-PAGE gel and detected with a mouse monoclonal antibody (Alexis Biochemicals). To detect the 35 kDa pro-caspase form of caspase-3, proteins were resolved in a 10% SDS-PAGE gel and detected with caspase-3 rabbit monoclonal antibody (Cell Signaling Technology). To detect the pro- (116 kDa) form of PARP, proteins were resolved in a 7.5% SDS-PAGE gel and detected with a rabbit monoclonal antibody (Cell Signaling).

In comparison with all three breast cancer cell lines tested, nHMECs infected with AAV2 did not show cleavage of initiator or executioner caspases tested (Figure 10), as would be expected from the inability of AAV2 to express Rep proteins or undergo active replication in nHMECs. Cumulatively, our results presented here suggest the ability of AAV2 to activate multiple pathways of apoptosis, and potentially necrosis, in a range of human breast cancer derived cell lines representing different grades of aggressiveness. However, regardless of the mode of cell death implemented, AAV2 infection cumulatively resulted in complete cell death.

### AAV2 Rep protein expression and apoptosis induction also results in deregulation of cell cycle check-point controls

**Figure 11:**
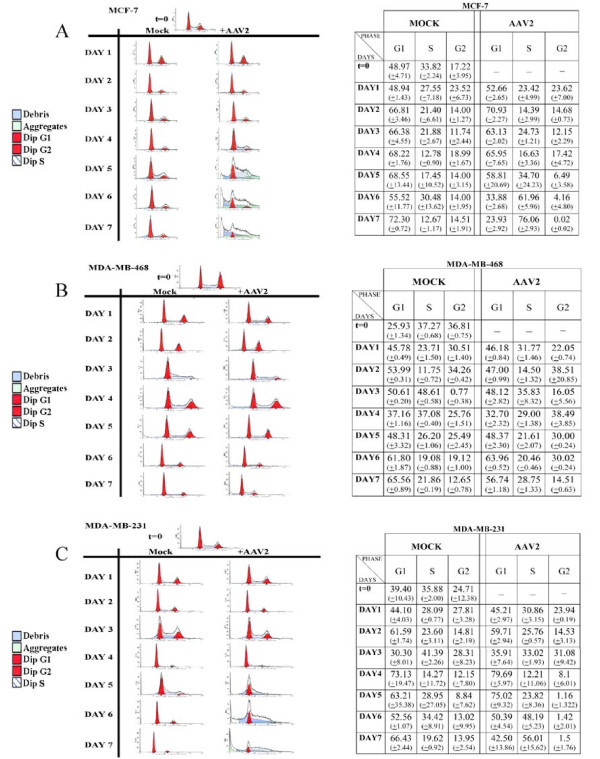
**Cell cycle progression and Fluorescence Activated Cell Sorting (FACS) analysis profiles of AAV2 infected breast cancer cell lines**. Cells were synchronized by trypsinization of 80% confluent cultures and plating at a density of 1 × 10^6 ^cells in E medium. The cells were incubated for 10 to 12 h at which point at least two-thirds of the cells are maximally synchronized in the G1 phase. This time point was designated time zero (t = 0). (A) MCF-7 (B) MDA-MB-468 and (C) MDA-MB-231 cells were infected with AAV2 at this point, and further cultured over a 7-day period. Both control and AAV2 infected cells were passaged 1:2 on day 2 and day 5. On each day, cells were harvested by trypsinization, washed with PBS, fixed in 70% ethanol, and stored at -20°C for 24 h. For performing FACS analysis, cells were resuspended in PBS containing 0.1% Triton X-100, 200 μg/ml DNase-free RNase A, and 100 μg/ml of propidium iodide for 30 min at 37°C. Flow cytometric analysis of 10^6 ^cells was carried out in a fluorescence-activated cell sorter, and the percentages of cells in theG1, S and G2/M phases of the cell cycle were determined using the Cell Quest program of Becton Dickinson. Data were analyzed with the Mod Fit LT program. For each cell line, FACS analyses were repeated three times. Results shown represent averages determined from three individual experiments (-, no data), with standard deviations presented in parentheses.

**Figure 12:**
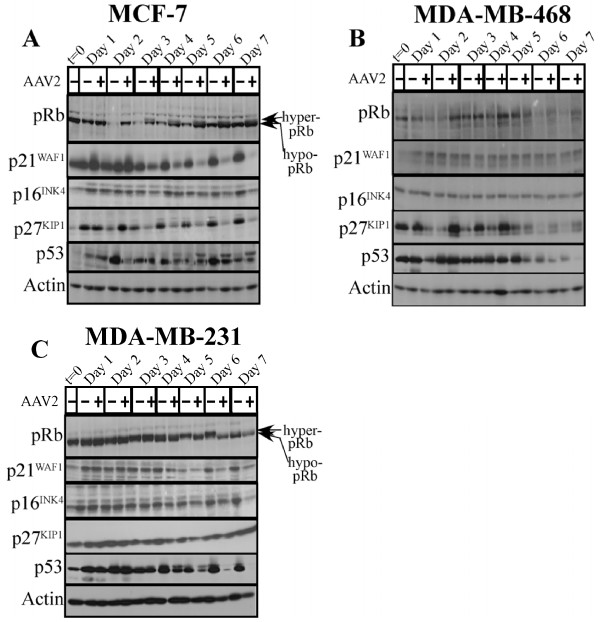
**AAV2 infection of different breast cancer cells and induction of apoptosis/cell death results in altered expression profiles of different cell cycle tumor suppressors proteins**. (A) MCF-7 (B) MDA-MB-468 and (C) MDA-MB-231 monolayer cell cultures were synchronized by trypsinization of 80% confluent cultures and plating at a density of 1 × 10^6 ^cells in E medium. The cells were incubated for 10 to 12 h at which point at least two-thirds of the cells are maximally synchronized in the G1 phase followed by infection with AAV2. This time point was designated time zero (t = 0). Cell pellets were collected each day over a 7 day period. Cells were passaged 1:2 on day 2 and day 5. Detection of cell cycle tumor suppressor proteins was performed by Western blotting. Total protein extracts were prepared as described previously [48]. Sixty micrograms of total protein extracts from AAV2 infected and control cells were resolved in SDS-polyacrylamide gel electrophoresis (PAGE) gels. The pRb protein hyperphosphorylated (Hyper-pRb) and hypophosphorylated (hypo-pRb) forms were detected using the rabbit polyclonal antibody (Santa Cruz). The p21^WAF1^, p27^KIP1 ^and p16^INK4 ^tumor suppressors were detecting using rabbit polyclonal antibodies (Santa Cruz) as described in Materials and Methods. The p53 protein was detected using a mouse monoclonal antibody (Oncogene).

Thus far, AAV2 mediated cell death failed to reveal any common mechanisms of apoptosis among the different breast cancer lines studied. Since mechanisms controlling cell proliferation and cell death are interconnected [70-72], we next examined pathways of cell cycle progression. To determine whether apoptosis induction in AAV2 infected breast cancer cells coincided with changes in cell cycle progression, we performed fluorescence-activated cell sorting (FACS) analysis and determined the percentage distribution of mock and AAV2 infected cells in G1, S, G2 and M phases. We observed a sustained increase in the percentage of AAV2 infected MCF-7 cells entering into S phase on day 4 to day 7 compared with controls (Figure 11A). Increased S phase entry occurred subsequent to Rep protein expression on day 3 in the AAV2 infected MCF-7 cells (Figure 3A). Taken together, these results suggested Rep protein mediated a breach of the G1/S check-point control which allowed for the increased S phase entry and progression (Figure 11A) and apoptosis induction (Figure 1A). On day 3, Rep protein expression in AAV2 infected MCF-7 cells (Figure 3) was correlated with steady-state pRb protein expression displaying both the hyper-phosphorylated (inactive) and hypo-phosphorylated form of this tumor suppressor (Figure 12A). The appearance of the hyper-phosphorylated species of pRb in AAV2 infected cells could have at least two simultaneous consequences on cell fate. First, pRb inactivation and E2F release could result in E2F transcription of S phase genes [73]. Second, DNA damage-induced pRb post-translational modification and inactivation could also promote E2F1 transcription of pro-apoptotic genes [74]. Therefore, in AAV2 infected MCF-7 cells, death could occur due to conflicting signals arising from a breach of G1/S checkpoints, followed by S phase entry and progression (compare Figure 11A and 12A), in the presence of damaged DNA (Figure 1A). On day 3, Rep protein expression in MCF-7 cells (Figure 3) also coincided with decreased expression of the CDK inhibitors p21^WAF1 ^and p27^KIP1 ^(Figure 12A), thereby establishing conditions resembling a cellular environment which is permissive for S phase entry [75]. Additionally, dropping cellular p21^WAF1 ^protein levels decreases the ability of cells to block apoptosis [76], and may act to prime the AAV2 infected MCF-7 cells for downstream events which culminate in apoptosis induction. We also determined levels of p53 protein expression, as in normal cells p53 is a transcriptional activator of p21^WAF1 ^[77]. In the AAV2 infected MCF-7 cells, significant changes in total p53 levels were not observed (Figure 12A). However, a slower migrating band was detected with the p53 antibody. This band could potentially represent phosphorylated species of p53 known to be induced upon activation of DNA damage signals [78]. These results are not unexpected since the MCF-7 cells harbor a normal p53 [79]. Finally, on day 5 to day 7, AAV2 infected MCF-7 cells undergoing apoptosis showed decreased levels of the p16^INK4 ^tumor suppressor protein compared with controls (Figure 12A). In contrast, control cells reached confluency and became contact inhibited (Figure 11A). The observed AAV2 mediated downregulation of multiple CDK inhibitors, p21^WAF1^, p27^KIP1 ^and p16^INK4^, could be a mechanism necessary to counteract cell cycle check-point regulated growth arrest in AAV2 infected cells and induction of apoptosis.

In the AAV2 infected MDA-MB-231 cells, apoptosis induction similarly coincided with increased S phase entry (Figure 11C), but at a much later time point (day 6) in the time-course compared to the virus infected MCF-7 cells (compare Figure 11A and 11C). However, in contrast to the AAV2 infected MCF-7 cells, increased S phase entry in AAV2 infected MDA-MB-231 cells was marked with the appearance of hypo-phosphorylated pRb (Figure 12C). Others have also reported the ability of Rep78 to promote pRb hypo-phosphorylation and a complete S phase arrest [40,80]. In the AAV2 infected MDA-MB-231 cells, S phase entry and apoptosis induction also coincided with decreased levels of p21^WAF1^, p16^INK4 ^and p53, but not p27^KIP1^, compared with controls (Figure 12C). Overall, AAV2 induced cell death in both the MCF-7 and MDA-MB-231 cell lines appeared to target cell cycle check-point controls which potentially mediate a breach in the G1/S cell cycle check-point, further promoting entry of these cells into S phase.

In contrast to the other two breast cancer cell lines, AAV2 infected MDA-MB-468 cells increasingly entered into G2 phase (day 3 to day 6) compared with uninfected cells (Figure 11B), which further coincided with apoptotic DNA laddering (Figure 1B). During this time frame, weak Rep protein expression could be observed on day 3 through day 6 in AAV2 infected cells (Figure 3B). On day 7, Rep78 expression was increased compared to day 6 and AAV2 infected apoptotic MDA-MB-468 cells displayed an increased percentage of cells with S phase DNA content compared with controls (Figure 11B). These results suggest that on day 7, increased movement out of G2 into S phase coincided with strong expression of Rep 78 on day 7 (Figure 3B). AAV2 regulated cell cycle progression in the infected MDA-MB-468 cells could not be correlated with significant changes with pRb phosphorylation and the protein was expressed in low levels (Figure 12B). Additionally, AAV2 infection of MDA-MB-468 cells did not induce significant changes in the total levels of p21^WAF1^, p16^INK4 ^and p53, but p27^KIP1 ^protein levels were significantly increased compared with controls (day 2 to day 7) (Figure 12B). Increased p27^KIP1 ^protein levels could potentially mediate increased accumulation of these cells in the G2 phase of the AAV2 infected cells undergoing apoptosis (compare Figure 11B and 12B). Our results suggest that AAV2 mediated G2 phase enrichment in the MDA-MB-468 cells was sufficient for apoptosis induction as determined from DNA laddering (Figure 1B), which occurred in the presence of low level Rep protein expression (Figure 3B).

The differences in the kinetics of Rep protein expression and cell death among the three AAV2 infected breast cancer cell lines appeared to synchronize with selective enrichment of S phase specific functions. A prominent requirement for G2 phase enrichment in the MDA-MB-468 cells coincided with apoptotic DNA laddering in the presence of low level Rep protein expression. Therefore, initiation of apoptosis could efficiently occur in S or G2 phases in the breast cancer cell lines tested. In the AAV2 infected MDA-MB-468 cells, Rep 78 expression was observed with clarity only on day 7 (Figure 3B) which also correlated with increased movement into S phase (Figure 11B). Therefore, it is possible that specific cellular functions which are conducive for abundant Rep protein expression generally become available upon S phase entry. Our results cumulatively suggest that AAV2 targeting and enhanced activation of key cell cycle check-points in G1/S, S and G2 phases could be a cellular mechanism for Rep protein synthesis, and further, could provide a priming mechanism for inducing apoptosis in the different breast cancer derived cell lines. The comparatively lower levels of Rep protein expression in AAV2 infected MDA-MB-468 cells may be one explanation as to why these cells arrest more in G2 than in S phase.

### AAV2 mediated manipulation of cell cycle check-points is marked by upregulation of c-Myc and Ki-67 protein levels

Thus far, our results show that AAV2 induction of apoptosis in the MCF-7 and MDA-MB-231 cells coincided with pronounced S phase entry, whereas an initial G2 phase enrichment followed by S phase entry was observed in the MDA-MB-468 cells. Increased breach of the G1/S check-point and movement of cells into S phase is indicative of implementation of cellular proliferation signals [81]. Likewise, increased G2 phase progression signals readiness of the cell to enter mitosis [82]. AAV2 targeted interference with mechanisms which control cell cycle check-points in cells undergoing DNA damage could be a trigger for apoptosis induction. Therefore, we reasoned that one or more signaling cascades upstream of cell cycle controls could mechanistically couple both increased S and G2 phase progression, proliferation and apoptosis induction.

**Figure 13:**
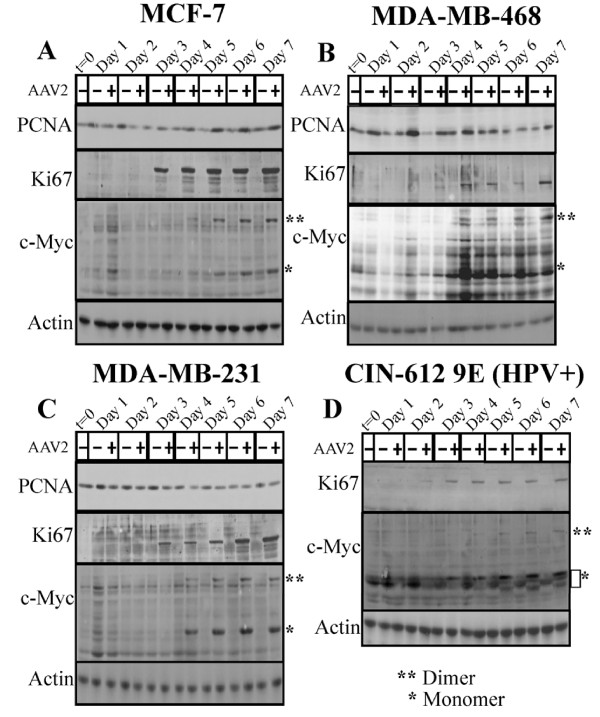
**AAV2 infection of different breast cancer cell lines and induction of apoptosis/cell death results in altered expression of proliferation markers PCNA, Ki67 and c-Myc**. (A) MCF-7 (B) MDA-MB-468 (C) MDA-MB-231 and (D) CIN-612 9E (HPV31b positive cervical cancer cells) monolayer cell cultures were synchronized by trypsinization of 80% confluent cultures and plating at a density of 1 × 10^6 ^cells in E medium. The cells were incubated for 10 to 12 h at which point at least two-thirds of the cells are maximally synchronized in the G1 phase followed by infection with AAV2. This time point was designated time zero (t = 0). Cell pellets were collected each day over a 7 day period. Cells were passaged 1:2 on day 2 and day 5. Detection of cell cycle proliferation proteins was performed by Western blotting. Total protein extracts were prepared as described previously [48]. Sixty micrograms of total protein extracts from AAV2 infected and control cells were resolved in SDS-polyacrylamide gel electrophoresis (PAGE) gels. The PCNA protein was detected using a rabbit polyclonal antibody (Santa Cruz). The Ki67 protein was detected using a rabbit polyclonal antibody (Santa Cruz). The c-Myc protein was detected using a rabbit polyclonal antibody (Santa Cruz).

In normal cells, entry of cells into the G1 phase and execution of the G1/S cell cycle check-point is dependent on multiple signaling mechanisms, a key regulator of which is c-Myc [81]. On the other hand, PCNA and Ki67 are markers of S phase proliferation [83,84]. We performed western blots to detect the expression of PCNA, Ki67 and c-Myc, in AAV2 infected breast cancer cell lines tested. PCNA expression was found to be increased in AAV2 infected MCF-7 and MDA-MB-468 cells compared with mock infected cells (day 1 to day 7) (Figure 13A and 13B), but was somewhat decreased in AAV2 infected MDA-MB-231 cells (Figure 13C). On the other hand, Ki-67 expression was increased in all the AAV2 infected breast cancer cells day 3 onwards (Figure 13), which is indicative of signals for proliferation but which coincided with decreased cell viability (Figure 2). Expression of c-Myc was greatly upregulated in all AAV2 infected breast cancer cells starting on day 4 (Figure 13A-C). Both the dimer- and monomer forms of c-Myc were detected in each cell line (Figure 13A-C). Our results suggest that AAV2 regulated expression and/or stabilization of c-Myc could serve to amplify proliferation signals which allow the breast cancer cells to bypass cell cycle checkpoint controls in the presence of damaged cellular DNA. On the other hand, c-Myc is also a well studied pro-apoptotic protein [85], which has the added potential to regulate DNA damage response upon imposition of genotoxic stress [86] mediated via the inherent endonuclease activity of AAV2 Rep78 and Rep68 proteins [80]. Thus, in the breast cancer cell lines, AAV2 regulation of c-Myc activation could potentially mediate simultaneous growth stimulatory (G1/S, S and G2 phase targeting), and growth inhibitory (apoptosis/necrosis associated DNA damage) signals which could represent a central unifying mechanism of AAV2 induced cell death in multiple breast cancer types. In addition, AAV2 infection also regulated increased c-Myc and Ki67 expression in apoptotic HPV positive cervical cancer lines (Figure 13D), suggesting that AAV2 implements common mechanisms of cell death in multiple grades of breast cancer cells as well as in HPV positive cervical cancer cells.

## Discussion

The current study investigates the mechanisms by which AAV2 induces apoptosis and/or cell death in the different breast cancer cell lines. Overall, the significant finding of this study was that while all three breast cancer cell lines used underwent cell death in response to AAV2, but not the primary breast epithelial cells, there did not appear to be a "universal" pathway underlying the demise of the three cancer lines. The underlying molecular mechanism could involve the ability of the AAV2 Rep proteins to push the cancer cells towards a proliferative phenotype, as indicated by the increased movement of cells into S (MCF-7 and MDA-MB-231 cells) and G2 phases (MDA-MB-468 cells), while simultaneously overcoming some DNA damage checkpoint regulatory controls. In normal cells, DNA damage signals activate checkpoint kinases (Chk) play a critical role in determining cellular responses to DNA damage by initiating cell cycle arrest, thus allowing for DNA repair [87]. The S, intra-S, and the G2-M phase checkpoints are predominantly regulated by Chk1, whereas Chk2 regulates the G1 phase checkpoint [87]. In the current study, addition of DNA damage via AAV2 genomic inverted terminal repeats [88], or Rep protein nuclease functions [80], could induce a significant level of cellular stress, particularly in cancer cells which are already compromised for DNA damage checkpoint functions [87]. By promoting the virus infected breast cancer cell lines to increasingly enter S and G2 phases in the presence of measurable DNA damage, potentially indicates the ability of AAV2 to interfere with Chk1 kinase functions. In a way, AAV2 action on the breast cancer cells may be similar to the use of DNA damage checkpoint kinase inhibitors which sensitize cancer cells to genotoxic chemotherapeutics, while non-cancerous cells are more resistant to such therapeutics due to the intact DNA damage checkpoint pathways.

### AAV2 induced c-Myc expression is a regulator of both cell proliferation and apoptosis

In the current study, we showed that multiple human breast cancer derived cell lines representing both low and high grades of aggressiveness, were permissive for AAV2 replication and Rep protein expression, ultimately resulting in 100% cell death. Common to all the breast cancer cell lines tested, AAV2 mediated induction of apoptosis coincided with upregulated expression of the c-Myc protein, a transcription factor which is both a regulator of cell proliferation as well as apoptosis [89]. Under normal conditions, c-Myc controls growth regulatory signals in early G1 and at the G1/S cell-cycle check-points [90]. However, activity of c-Myc is also deregulated in multiple cancers causing uncontrolled cellular proliferation [91]. It has been shown that c-Myc regulated processes of mitogenesis and apoptosis are controlled by activation of disparate cellular machineries [92]. Diverse stress-related signals such as growth factor deprivation, hypoxia and exposure to genotoxic agents have been shown to trigger c-Myc dependent apoptosis [93]. Acute activation of c-Myc sensitizes cells to apoptosis by inducing cytochrome *c *release from the mitochondria through activation of the pro-apoptotic members of the Bcl-2 family in cells deprived of growth factors [94]. Additionally, overexpression of c-Myc was shown to accelerate the apoptotic response by sensitizing cells to pro-apoptotic insults [95].

### pRb phosphorylation status as a dual marker of AAV2 regulated proliferation and cell death

AAV2 induced apoptosis was correlated with increased S-phase entry in the MCF-7 and MDA-MB-231 cells which is likely due in part to the observed increased c-Myc expression. Ectopic expression of c-Myc has been shown to induce S phase entry in quiescent cells, observations which first suggested that c-Myc can play a role in G1/S transition, similar to that observed for E2F1 [90]. However, the increased S phase entry observed had cell specific consequences on the status of pRb phosphorylation. In the AAV2 infected MCF-7 cells, pRb protein expression was stabilized displaying both hyperphosphorylated (inactive) and hypophosphorylated (active) forms of the protein. Normally, pRb inactivation and E2F release is required for E2F transcription of S-phase genes [73]. However, DNA-damage-induced pRb acetylation/inactivation promotes E2F1 transcription of proapoptotic genes [74]. In contrast, DNA damage-induced Chk2-mediated phosphorylation of pRb leads to E2F sequestration and transcriptional inhibition of proapoptotic genes [96]. In the current study, we demonstrated the ability of AAV2 to catalyze S-phase entry in cycling MCF-7 cells in the presence of pRb in both its active and inactive forms, which could represent a novel trigger for the induction of apoptosis. In MCF-7 cells infected with AAV2, death could occur due to conflicting signals arising from a breach of G1/S checkpoints, followed by S-phase entry and progression (mediated by hyperphosphorylated pRb), simultaneous with the transcription of genes related to differentiation and grown arrest (activated by hypophosphorylated pRb). On the other hand, AAV2 infection of MDA-MB-231 cells induced increased entry into S phase and displayed hypophosphorylated pRb. Induction of necrosis as a mode of cell death and associated energy depletion [97] could result in the failure to maintain phosphorylated species of pRb. Others have reported the ability of Rep78 to promote pRb hypophosphorylation and a complete S-phase arrest [40,80] mediated via Rep78 binding to CDC25A and preventing access to its substrates, CDK2 and CDK1 [40,80].

### AAV2 modulates cellular tumor suppressor protein levels to induce movement through S and G2 phases

It is notable that AAV2 triggered S-phase entry in MCF-7 and MDA-MB-231 cells occurred in the presence of decreased levels of CDK inhibitors p21^WAF1^, p27^KIP1 ^and p16^INK4^, conditions which would be expected to enhance entry into S phase, and which could be a mechanism whereby AAV2 competes with the cancer cells for cellular factors for its own transcription. AAV2 mediated downregulation of p21^WAF1 ^levels may act to prime the breast cancer cells for downstream events which culminate in apoptosis. In support of our observations, antisense oligonucleotide to p21^WAF1 ^was shown to attenuate p21^WAF1 ^levels and induce apoptosis in MCF-7 cells [98]. On the other hand, the MDA-MB-468 cells infected with AAV2 increasingly entered into G2. Other reports indicate that c-Myc expression could also cause G2/M arrest and mediate apoptosis induction [99]. In normal cells, intact p53 and p21^WAF1 ^proteins respond to DNA damage signaling by sustaining a G2-phase arrest which is a protective cellular mechanism for allowing DNA repair before proceeding into mitosis [100]. However, apoptotic DNA laddering was observed in AAV2 infected MDA-MB-468 cells which were enriched in the G2 phase of the cell cycle in the presence of increased levels of p27^KIP1^, which in normal cells has the potential to mediate growth arrest [101].

### AAV2 regulated cell death does not result from activation of common pathways of apoptosis

c-Myc is a potent inducer of apoptosis [85] and was clearly expressed in all the AAV2 infected breast cancer cell lines. However, our results showed that the final stages of AAV2 induced cell death in the multiple breast cancer cell lines was not due to activation of common pathways of apoptosis. A major difference among the different breast cancer cell lines used was the expression of caspase-3. In the MCF-7 cells, the loss of caspase-3 has been shown to correlate with resistance to drug-induced apoptosis in breast cancer cells [102]. In the current study, induction of caspase-independent apoptotic cell death in MCF-7 cells potentially resulted from AAV2-regulated pro-apoptotic signaling at the level of the mitochondria resulting in the release of mitochondrial factors such as Apoptosis Inducing Factor (AIF) from the intermembrane space, its translocation to the nucleus, and subsequent triggering of nuclear apoptosis by facilitating DNA fragmentation and chromatin condensation [103]. We also observed AAV2 specific PARP cleavage in a caspase-independent manner, which could be regulated by calpain, a Ca^2+^-dependent intracellular cysteine protease, a finding also previously reported in oridonin treated MCF-7 cells [65]. In contrast, although the MDA-MB-231 cell line expresses caspase-3, AAV2 infection of this cell line potentially resulted in a mixture of caspase-dependent and -independent forms of cell death. AAV2 mediated simultaneous cleavage of both caspase-8 and caspase-9 suggesting activation of the extrinsic and intrinsic pathways of apoptosis respectively, however downstream activation of executioner caspases could not be clearly defined due to non-specific degradation of both their uncleaved and cleaved forms. Since PARP cleavage was not observed in the AAV2 infected MDA-MB-231 cells, our results indirectly suggested that the activity of executioner caspases could have been inhibited in virus infected cells. Others have reported that mechanistic inactivation of caspases causes a shift from apoptosis to cell death consisting of mixed necrotic/apoptotic features, or full-blown necrosis [104]. Activation of necrosis is a default pathway of cell death which is unmasked when the apoptotic pathway is inhibited [105]. Failure to observe PARP cleavage in the AAV2 infected MDA-MB-231 cells not only highlights absence of caspase activation, but also potentially suggests activation of necrosis as mode of cell death and agrees with observations in other published reports [106]. In cells undergoing extensive DNA damage, cellular response to repair the DNA results in hyperactivation of PARP-1, which subsequently results in the depletion of cytosolic NAD^+^, and its precursor ATP, leading to necrotic cell death via energy failure [97]. Additionally, since the completion of apoptosis is absolutely dependent on the presence of ATP, upon its cellular depletion, cell death switches from apoptosis to necrosis [107]. Thus, caspase-9 cleavage in AAV2 infected MDA-MB-231 cells undergoing DNA degradation indicates activation of apoptosis but failure to cleave PARP is further indicative of the switch to necrosis. In contrast, among the three cell lines, only the AAV2 infected MDA-MB-468 cell line resulted in caspase-9 dependent apoptosis which was clearly correlated with caspase-3 and PARP cleavage.

Cumulatively, our results suggest that AAV2 regulated Rep protein expression leads to cell death. Our present findings have relevance for understanding the mechanisms of AAV2 induction of complete cell death in breast cancer cells as well as studying mechanisms of resistance to breast cancer therapy. By regulating the expression of c-Myc, AAV2 potentially targets a central protein which regulates multiple processes of cell survival, cell cycle and apoptosis [90] in the multiple breast cancer lines. An additional c-Myc regulated effector acting upstream of the mitochondrial apoptotic machinery is Ataxia telangiectasia mutated (ATM)-dependent DNA damage checkpoint responses in response to genotoxic stress [86]. Incidentally, AAV2 Rep 78 and Rep 68 mediated nicking of cellular chromatin has also been shown to induce a DNA damage response via activation of ATM [80]. Thus, AAV2 could serve to further sensitize c-Myc regulated pro-apoptotic functions.

## Conclusions

The cellular links between cell survival, cell death and cell cycle have been recently reviewed [108]. In the current study, our results highlight the ability of AAV2 to regulate cell cycle and activate multiple routes of apoptotic cell death, which could provide insights into understanding the cellular mechanisms of drug resistance and global targeting of breast cancer. On the other hand, AAV2 infected nHMECs failed to undergo productive AAV2 replication and the failure of AAV2 to productively infect normal epithelial cells could be clinically advantageous and potentially portrays AAV2 as an ideal biological therapeutic. Our results suggest the potential for AAV2 to be developed as therapeutics for multiple types of breast cancer. Further, understanding the molecular mechanisms central to AAV2 targeting of cell death pathways in breast cancer derived cell lines will help in the design of more effective therapeutics irrespective of the type and grade of breast cancer.

## List of Abbreviations

AAV2: Adeno-Associated Virus Type 2; HPV: Human Papillomavirus; nHMECs: Normal human mammary epithelial cells; FACS: Fluorescent Activated Cell Sorting; MOI: Multiplicity of Infection; ERα: Estrogen Receptor α; GFP: Green Fluorescent Protein

## Competing interests

The work described in this manuscript is part of a pending patent applied for by The Pennsylvania State University Intellectual Property Office.

## Authors' contributions

SA and CM conceptualized the experimental design and drafted the manuscript. SA performed all experiments. BSB performed the cell cycle western blots and provided critical evaluation of the manuscript. MJC and MI analyzed and interpreted the data. AT performed the southern blots. All authors read and approved the final manuscript.
